# *In Vivo* Characterization of Cortical and White Matter Microstructural Pathology in Growth Hormone-Secreting Pituitary Adenoma

**DOI:** 10.3389/fonc.2021.641359

**Published:** 2021-04-12

**Authors:** Taoyang Yuan, Jianyou Ying, Chuzhong Li, Lu Jin, Jie Kang, Yuanyu Shi, Songbai Gui, Chunhui Liu, Rui Wang, Zhentao Zuo, Yazhuo Zhang

**Affiliations:** ^1^ Beijing Neurosurgical Institute, Capital Medical University, Beijing, China; ^2^ Department of Neurosurgery, Beijing Tiantan Hospital, Capital Medical University, Beijing, China; ^3^ State Key Laboratory of Brain and Cognitive Science, Institute of Biophysics, Chinese Academy of Sciences, Beijing, China; ^4^ University of Chinese Academy of Sciences, Chinese Academy of Sciences, Beijing, China; ^5^ CAS Center for Excellence in Brain Science and Intelligence Technology, Chinese Academy of Sciences, Beijing, China; ^6^ Beijing Institute for Brain Disorders Brain Tumour Center, China National Clinical Research Center for Neurological Diseases, Key Laboratory of Central Nervous System Injury Research, Beijing, China

**Keywords:** growth hormone-secreting pituitary adenoma, neurite orientation dispersion and density imaging (NODDI), myelin imaging, DTI (diffusion tensor imaging), neuropsychological dysfunction

## Abstract

**Background:**

The growth hormone (GH) and insulin-like-growth factor 1 (IGF-1) axis has long been recognized for its critical role in brain growth, development. This study was designed to investigate microstructural pathology in the cortex and white matter in growth hormone-secreting pituitary adenoma, which characterized by excessive secretion of GH and IGF-1.

**Methods:**

29 patients with growth hormone-secreting pituitary adenoma (acromegaly) and 31 patients with non-functional pituitary adenoma as controls were recruited and assessed using neuropsychological test, surface-based morphometry, T1/T2-weighted myelin-sensitive magnetic resonance imaging, neurite orientation dispersion and density imaging, and diffusion tensor imaging.

**Results:**

Compared to controls, we found 1) acromegaly had significantly increased cortical thickness throughout the bilateral cortex (pFDR < 0.05). 2) T1/T2-weighted ratio in the cortex were decreased in the bilateral occipital cortex and pre/postcentral central gyri but increased in the bilateral fusiform, insular, and superior temporal gyri in acromegaly (pFDR < 0.05). 3) T1/T2-weighted ratio were decreased in most bundles, and only a few areas showed increases in acromegaly (pFDR < 0.05). 4) Neurite density index (NDI) was significantly lower throughout the cortex and bundles in acromegaly (pTFCE < 0.05). 5) lower fractional anisotropy (FA) and higher mean diffusivity (MD), axial diffusivity (AD) and radial diffusivity (RD) in extensive bundles in acromegaly (pTFCE < 0.05). 6) microstructural pathology in the cortex and white matter were associated with neuropsychological dysfunction in acromegaly.

**Conclusions:**

Our findings suggested that long-term persistent and excess serum GH/IGF-1 levels alter the microstructure in the cortex and white matter in acromegaly, which may be responsible for neuropsychological dysfunction.

## Introduction

Acromegaly, mostly caused by growth hormone-secreting pituitary adenoma, is an endocrine and metabolic disease characterized by excessive secretion of growth hormone (GH) and concomitant increases in insulin-like-growth factor 1 (IGF-1) levels, leading to progressive somatic disfigurement and organ overgrowth (1, 2). Some studies have also demonstrated that acromegaly results in neurologic complications, such as peripheral neuropathy and cognitive dysfunction (3, 4). In most cases, acromegaly is caused by a growth hormone-secreting pituitary adenoma (2). Untreated patients with acromegaly have a reduced life expectancy due to cardiovascular, cerebrovascular disease, and respiratory diseases (5, 6). The clinical features of acromegaly develop in a chronic and insidious manner, such that the average time from initial onset of the disease to diagnosis is typically several years or even longer (7).

Previous studies have demonstrated that GH and IGF-1 play critical roles in the peripheral nervous system (PNS) and central nervous system (CNS), including in the process of brain growth, development, and myelination, as well as in procognitive and neuroprotective actions, neurogenesis processes and plasticity, and their impact on the nervous system throughout the entire lifespan (8–11). For example, clinical studies have demonstrated that patients with GH deficiency exhibit cognitive impairment and that treatment with recombinant GH can successfully improve cognitive functions (12, 13). In mice, GH or IGF-1 gene knock-out results in reduced brain size, loss of myelination and specific parvalbumin-containing neurons, and cognitive impairments (14, 15). Serum levels of GH and IGF-1 decrease with aging, and lower blood GH/IGF-1 levels were associated with poor cognitive ability in healthy older adults (10, 16). To date, however, the impact of long-term and persistent high levels of serum GH/IGF-1 on cortical and white matter microstructures such as myelin, axons and dendrites, in acromegalic patients have also been largely remained unknown.

In our previous study, we found that higher serum GH/IGF-1 levels significantly increased the volume of brain tissue (grey matter (GM) and white matter (WM) at the expense of cerebrospinal fluid volume (CSFV) and that GH/IGF-1 levels were associated with GM volume and CSFV, but not white matter volume, in acromegalic patients (17). In the current study, 29 active acromegalic patients (growth hormone-secreting pituitary adenoma) and 31 controls (patients with non-functional pituitary adenoma) were recruited to characterize the cortical and white matter microstructural pathology in acromegalic patients. Furthermore, we explored whether higher serum GH/IGF-1 levels could impair cognitive function and its possible mechanisms in acromegalic patients.

## Materials and Methods

### Participants

Thirty-two patients with active acromegaly (growth hormone-secreting pituitary adenoma) who underwent transnasal endoscopic surgery at the Department of Neurosurgery, Beijing Tiantan Hospital, from 2018 to 2019 were enrolled in the study. The inclusion criteria for the acromegalic patients were an age of 18–60 years and a diagnosis of active acromegaly needing surgical therapy. Acromegaly had been diagnosed by the presence of relevant clinical signs, increased serum GH and IGF-I levels, and/or failure of serum GH to be suppressed below 1μg/l after a 75-g oral glucose load. The control group was represented by 31 patients with non-functional pituitary adenoma to avoid the effect of the tumor occupying. The exclusion criteria for both groups included (1) a history of stroke, cerebral trauma, or other intracranial space-occupying lesions; (2) major psychiatric disorders, alcohol abuse or nicotine abuse; and (3) an inability to complete the MRI examinations or unsatisfactory imaging data. In the acromegaly group, three patients were excluded due to unsatisfactory imaging data in the process of imaging data analysis. Finally, only 29 patients were included in the acromegaly group.

The study was approved by the Beijing Tiantan hospital ethics committee. All procedures followed were in accordance with the ethical standards of the responsible committee on human experimentation and with the Helsinki Declaration of 1975, as revised in 2008. Informed consent was obtained from all patients for being included in the study.

### Neuropsychological Test

Montreal Cognitive Assessment (MoCA) (18), Digit Symbol Substitution Test (DSST) (19), Beck Depression Inventory (BDI) (20), and Self-Rating Anxiety Scale (SAS) (21) were used to assess the cognitive performance and mood in all subjects prior to surgery.

#### Biochemical Evaluation

The serum GH level in all subjects and serum IGF-1 level in all acromegalic patients were measured prior to surgery. The serum value of GH/IGF-1 in venous blood samples which collected between 06:00 a.m. and 10:00 a.m. following 10–12 h of fasting were measured using the IMMULITE 2000 immunoassay system (Siemens, Germany). To correct the effect of age and sex, serum IGF-1 and GH levels were calculated as follows: serum IGF-1 or GH value/95th percentile of the age- and sex-adjusted normal range (22).

#### Image Acquisition

MRI data were acquired at a 3T Siemens Prisma MRI scanner (Siemens Healthineers, Erlangen, Germany) with the same protocol in all subjects prior to surgery using a commercial 64-channel head coil, including (1) T1-weight image was acquired through a three-dimensional (3D) sagittal magnetization-prepared rapid acquisition gradient-echo sequence (224 slices; inversion time (TI)/echo time (TE)/repetition time (TR) = 1000/2.22/2400 ms; flip angle = 8°; bandwidth = 220 Hz/px; data matrix = 320 × 300; field of view (FOV) = 256 × 240 mm^2^ with a resolution of 0.8 mm isotropic voxels). (2) The 3D T2w images were acquired using the Siemens 3D SPACE sequence with same position as MPRAGE (spatial resolution 0.8 × 0.8 × 0.8 mm^3^, 224 slices, TR/TE = 3200/563 ms, data matrix = 320 × 300, FOV = 256 × 240 mm^2^). (3) Diffusion weighted images were acquired using a 2D spin-echo single-shot multiband echo-planar imaging (EPI) sequence with a multiband factor of 3 and a monopolar gradient pulse: slice thickness = 1.5 mm without gap, 100 axial slice coverage of the whole brain, TR/TE= 3500/86 ms, FOV= 210 × 210mm^2^, acquisition matrix = 140 × 140, voxel= 1.5 × 1.5 × 1.5 mm^3^. Another diffusion session included only b0 image collected in opposite (posterior-to-anterior) phase-encoding polarities. In our study, gradient tables published in the Connectome Coordination Facility protocol by the Human Connectome Project (HCP) group were used. In each gradient table, there were either 92 diffusion weighted directions and 7 b=0 s/mm^2^ acquisitions (b0 images). Diffusion weighting was composed of 2 shells of b=1000 and 2000 s/mm^2^, with nearly equivalent numbers of acquisitions distributed on each shell.

### Surface-Based Morphometry (SBM) Analysis

SBM was used for the cortical analysis, performed with CAT12 (http://dbm.neuro.uni-jena.de/cat), a Statistical Parametric Mapping 12 (SPM12, University College London, London, UK, http://www.fil.ion.ucl.ac.uk/spm) extension with the default pipeline in the MATLAB environment (MATHWORKS, California, USA). The SBM analysis uses a fully automated method that allows the measurement of cortical thickness and reconstruction of the central surface in one step and performs topological correction and spherical mapping for inter-subject alignment and spherical registration (23, 24). The image analysis procedure was performed according to the CAT12 manual. In our study, cortical thickness was compared between the acromegaly and controls. The statistical analysis was conducted using SPM12 and the CAT12 extension. Significance was determined using a positive false discovery rate (pFDR) < 0.05.

### Myelin-Sensitive Imaging

Determination of the T1/T2-weight ratio is a new method for non-invasive myelin-sensitive imaging *in vivo* (25). T1- and T2-weighted image data processing was conducted to obtain a myelin-sensitive contrast using the MR Tool-Multimodal Mapping extension (v.1.2, http://www.fil.ion.ucl.ac.uk/spm/ext) for SPM12. The difference in the T1/T2-weight ratio between the acromegaly and controls was assessed using two-sample t tests and significant group differences were adjusted for multiple comparisons using a positive false discovery rate (pFDR) < 0.05.

### Diffusion MRI Data Preprocessing and Analysis

Both single- and multi-shell data were pre-processed, including head motion correction, eddy current correction, and non-brain structures were removed using the FMRIB Software Library (FSL) (www.fmrib.ox.ac.uk/fsl). The NODDI three-compartment model including intracellular, extracellular, and CSF compartments was estimated using pre-processed data from the b = 1000 s/mm^2^ and b = 2000 s/mm^2^ diffusion-weighted data by Accelerated Microstructure Imaging *via* Complex Optimization (AMICO, https://github.com/daducci/AMICO/). The main parameters derived from NODDI are (1) the neurite density index (NDI), which reflects the packing density of axons or dendrites; (2) the neurite orientation dispersion index (ODI), which reflects the spatial organization of the axons; and (3) the volume fraction of isotropic water molecules (Viso), which reflects the proportion of free water (i.e., CSF) in a voxel. Finally, NOODI-derived maps of the intracellular volume fraction (NDI), isotropic volume fraction (Viso), and orientation dispersion index (ODI) were computed. Pre-processed data from the b = 1000 s/mm^2^ diffusion-weighted data were used to compute 3D maps of fractional anisotropy (FA), mean diffusivity (MD), axial diffusivity (AD) and radial diffusivity (RD). Individual NODDI and DTI metric maps were then spatially normalized to the group-specific template for subsequent Tract-Based Spatial Statistic (TBSS) (26).

NODDI metrics in GM were analyzed with Gray Matter-Based Spatial Statistics (GBSS), which is a statistical technique that adapts the TBSS framework to allow voxel-wise analysis (26). The processing steps for GBSS were performed according to previous studies (27, 28). Briefly, the GM fractional mapping in the original diffusion space was obtained by subtracting the CSF fraction maps (NODDI VISO parameter maps) and WM fraction maps (estimated from FA maps using the Atropos segmentation tool (29) in Advanced Normalization Tools [ANTs] v2.1.0) from 1 in each voxel. Next, to increase tissue contrasts and enhance between-subject registration steps, each tissue segmentation map was multiplied by their corresponding contrast (CSF=0, WM=1, GM=2) and summed to generate pseudo-T1-weighted images with similar contrast to T1-weighted images. Then, generated pseudo-T1-weighted images from all subjects were applied to generate a subject-specific template using the corresponding script in ANTs. Meanwhile, NODDI parameter maps (NDI and ODI) and GM fraction maps in native diffusion space were nonlinearly warped to the subject-specific template using the estimated warp fields. In addition, mean GM image was generated by averaging the GM fraction maps in all subjects and was then skeletonized using the tbss_skeleton tool in FSL. Finally, The NDI and ODI maps were projected from local voxels with the greatest GM fraction in the template space onto the GM skeleton. The final GM skeleton was thresholded to retain only the voxels with GM fraction > 0.65 in > 70% of the subjects.

For the TBSS and GBSS analyses, two-sample t tests were performed between the acromegaly and controls in FSL using permutation testing (n = 1,000) and threshold-free cluster enhancement (TFCE) was used for multiple comparison correction at p < 0.05. Age, sex, and education were included as covariates.

### Region of Interest (ROI) Analysis

To interrogate microstructural pathological alterations in specific brain regions and fiber bundles caused by long-term persistent excess serum GH/IGF-1 levels in acromegaly, we performed ROI analyses with both NODDI and DTI metrics. FSL’s plugin ‘AutoPtx’ was used to create subject-specific probabilistic representations of white matter bundles after automatic probabilistic fiber tractography. The mean values for the DTI and NODDI metrics for white matter were extracted from the 27 major white matter tracts, and mean values for the NODDI metrics in the cortex were extracted using the Montreal Neurological Institute (MNI) structural atlas. The group differences in DTI and NODDI metric values between the acromegaly and control groups were performed using two-sample t tests. A two-tailed P < 0.05 was considered statistically significant.

### Statistical Analysis

Line correlation was performed to assess the potential relationship between serum GH/IGF-1 level and T1/T2-weight ratio, NODDI and DTI metrics in the FSL, correcting for covariance (age, sex, and education). Statistical analysis for non-imaging data was performed in SPSS Statistics software (version 25, IBM Inc, Chicago, USA). Partial correlation was performed to assess the potential relationship between neuropsychological test and NODDI and DTI metrics, correcting for covariance (age and education). Group-differences in nominal variables were tested with Chi squared test. Unpaired t-test was applied to compare the continuous variables between groups. A two-tailed P < 0.05 was considered statistically significant.

## Results

### Participant Characteristics

The final analysis comprised 29 acromegalic patients and 31 patients with non-functional pituitary adenoma as the controls. The detailed demographic and clinical characteristics of the participants are summarized in **Table 1**. All patients with acromegaly were further confirmed to be GH positive by postoperative pathology. In our study, there were no significant differences in terms of age, sex, or education between the two groups. Means and standard deviations of the neuropsychological test scores in the two groups are also shown in **Table 1**. Overall, the acromegalic patients performed worse than the controls on the MoCA, DSST, and SAS tests. There was no significant difference in BDI scores between the two groups.

**Table 1:** Demographic and clinical characteristics of the participants.

Characteristic	Acromegaly	Controls	P-value
Numbers	29	31	NA
Age (mean ± SD, years)	41.3 ± 9.5	42.4 ± 9.7	0.66^a^
Sex (M/F)	14/15	18/13	0.45^b^
Education (mean± SD, years)	13.1 ± 2.8	13.4 ± 2.6	0.69^a^
Handedness, R/L, n	28/1	28/3	0.33^b^
Course of disease (months)	43.6 ± 40.9	14.8 ± 25.6	0.0017^a^
Normalized serum GH	3.9 ± 3.5	0.1 ± 0.1	<0.0001^a^
Normalized serum IGF-1	2.9 ± 0.9	NA	NA
Therapy	surgery	surgery	NA
Pathology	GH (+)	GH (-)	NA
MoCA	24.7 ± 3.0	28.9 ± 0.9	<0.0001^a^
DSST	48.1 ± 12.0	60.1 ± 10.3	=0.0001^a^
BDI	7.2 ± 6.1	7.1 ± 7.7	0.95^a^
SAS	38.3 ± 7.2	29.6 ± 6.1	<0.0001^a^

a Unpaired t-test, two-sided.

b chi squared test, two-sided.

### Surface-Based Cortical Thickness Analysis

**Figure 1:**
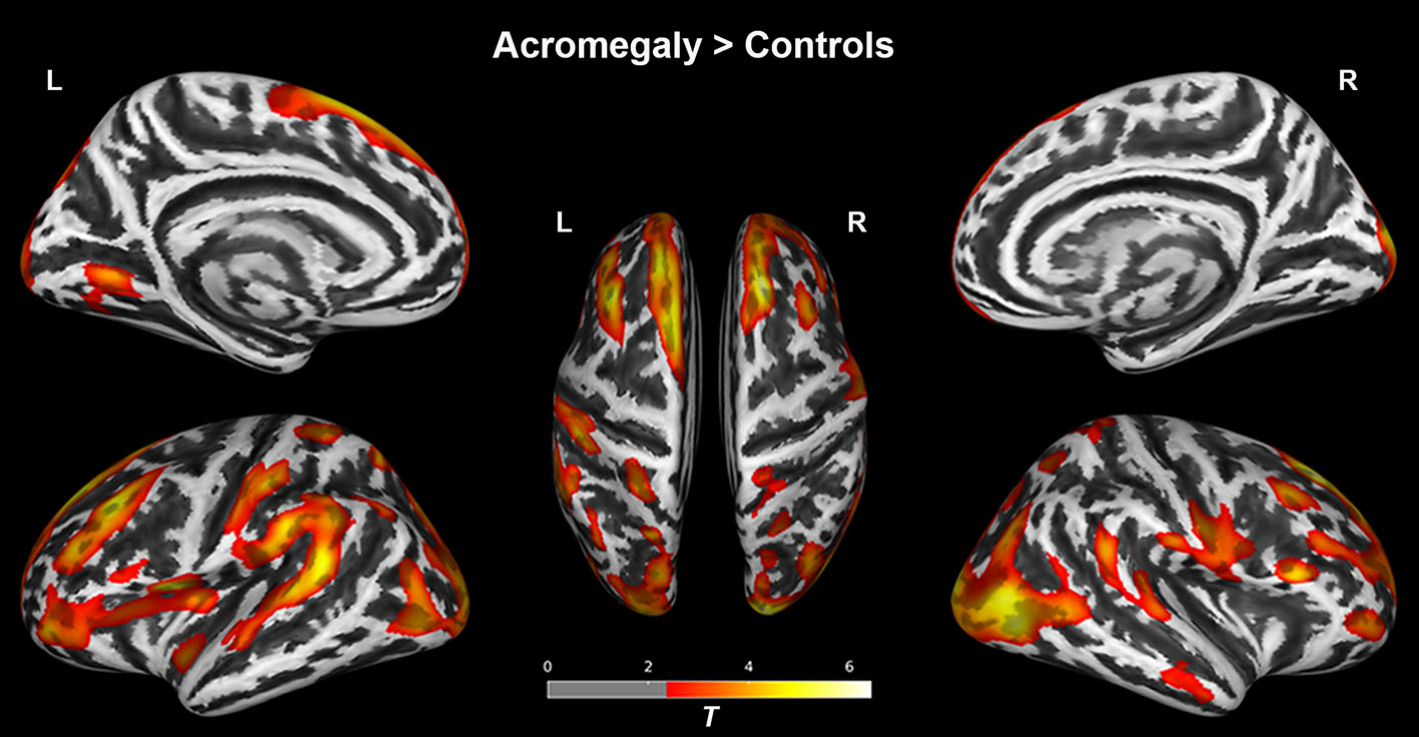
Results of the surface-based morphometry (thickness) analysis between acromegaly and controls. Red-yellow indicates the cortical thickness with significantly increased in acromegaly compared to controls. (pFDR < 0.05). L, left hemisphere; R, right hemisphere.

As shown in **Figure 1**, compared with the controls, the acromegaly had significantly increased cortical thickness in the bilateral superior and middle frontal regions, bilateral pre/postcentral gyri, bilateral inferior parietal regions, bilateral superior temporal gyri, and bilateral occipital lobe. No regions with significantly lower cortical thickness in the acromegaly relative to the controls were found.

### Myelin-Sensitive Imaging in the Cortex and White Matter

**Figure 2:**
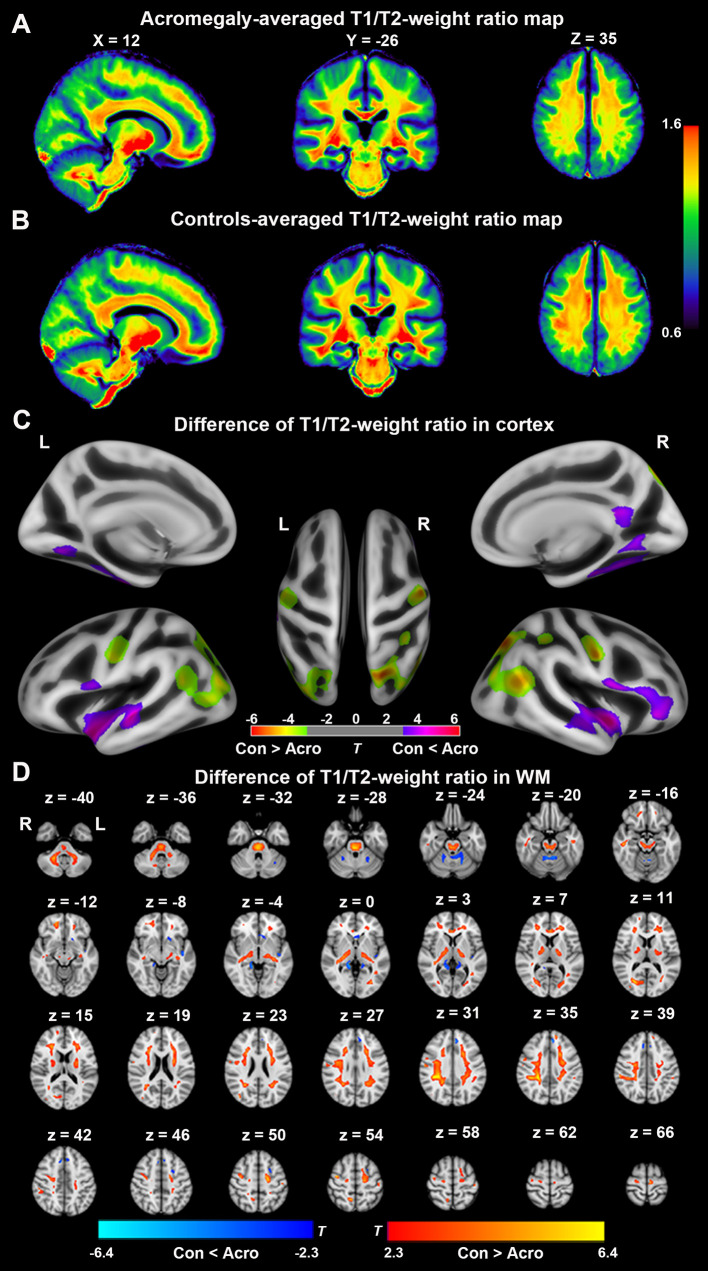
Difference of T1/T2-weight ratio in cortex and white matter (WM) between acromegaly and controls. Average T1/T2-weighted ratio maps from acromegaly **(A)** and controls **(B)**. **(C)** Green-yellow indicates the T1/T2-weight ratio in cortex was significantly decreased in acromegaly compared to controls (pFDR < 0.05). Violet-red indicates the T1/T2-weight ratio in GM was significantly increased in acromegaly compared to controls (pFDR < 0.05) **(D)** Red-yellow indicates the T1/T2-weight ratio in WM was significantly decreased in acromegaly compared to controls (pFDR < 0.05). Blue indicates the T1/T2-weight ratio in WM was significantly increased in acromegaly compared to controls (pFDR < 0.05). Con, controls; Acro, acromegaly.

Whole-brain group-averaged T1/T2-weight ratio maps are displayed in **Figures 2A, B**. In the cortex, as shown in **Figure 2C**, compared with the controls, acromegaly had significantly reduced T1/T2-weight ratio in the bilateral occipital cortex and pre/postcentral gyri and significantly increased T1/T2-weight ratio in the bilateral fusiform, bilateral insular, and bilateral superior temporal gyri (pFDR < 0.05). In the white matter, as shown in **Figure 2D**, compared with the controls, the acromegalic patients had widespread significant reductions in T1/T2-weight ratio, suggesting reductions in myelin content, and there were significant increases in T1/T2-weight ratio in only a few regions in the acromegalic patients compared with the controls.

### Diffusion Metrics in Cortex and White Matter

**Figure 3:**
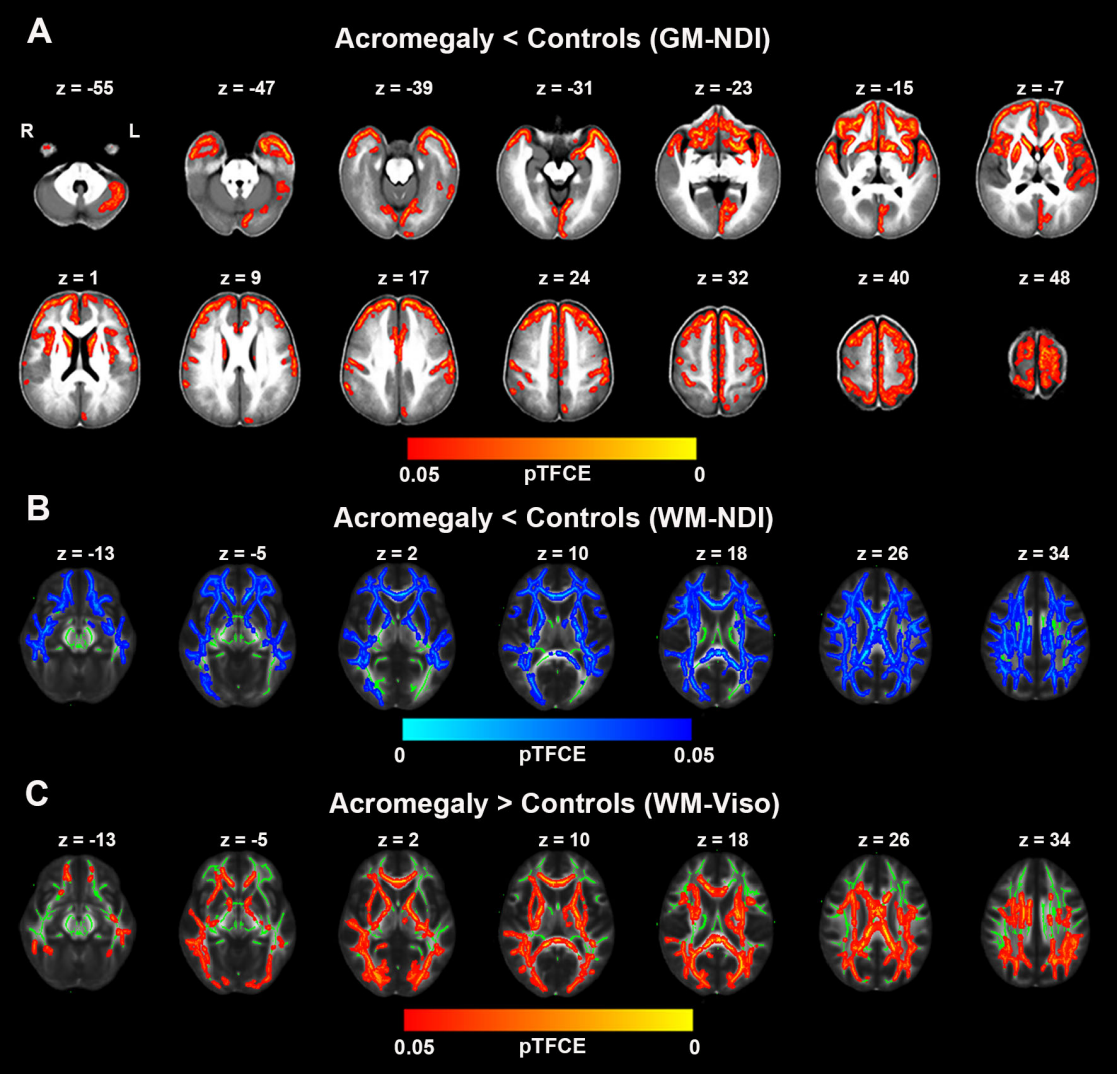
Microstructural pathological alterations in NODDI metrics in cortex and white matter in acromegaly. **(A)** showed lower neuritic density index (NDI) in acromegaly relative to controls (Red-yellow, pTFCE < 0.05) in gray matter–based spatial statistics (GBSS). **(B)** showed significant reductions in NDI across the whole brain white matter in acromegaly compared to controls (Blue, pTFCE < 0.05) in tract–based spatial statistics (TBSS). **(C)** showed significant increases in Viso across the whole brain white matter in acromegaly compared to controls (Red-yellow, pTFCE < 0.05) in tract–based spatial statistics (TBSS). GM, grey matter; WM, white matter.

GBSS was used to investigate cortical microstructural alterations from NODDI metrics between acromegaly and controls. Compared to controls, as shown in **Figure 3A**, acromegaly demonstrated significantly lower GM-NDI in widespread brain regions (pTFCE < 0.05). No significant differences in GM-ODI were observed between the acromegaly and controls.

We also investigated the white matter microstructural alterations in NODDI metrics using TBSS. Acromegaly showed significantly lower WM-NDI in many white matter fiber bundles than the controls (**Figure 3B**). There were no regions with significant differences in ODI between the acromegaly and the controls. With respect to Viso, there are significant increases in many clusters across the mean FA skeleton in the acromegaly compared to controls (**Figure 3C**).

**Figure 4:**
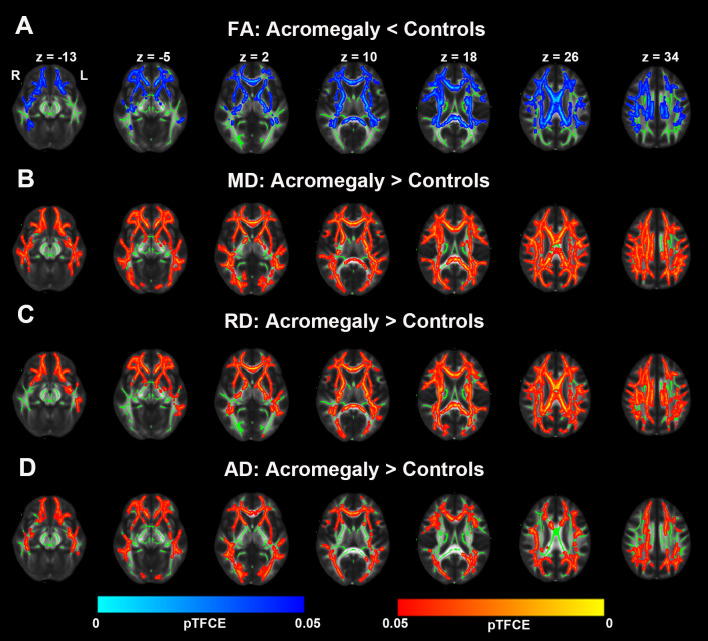
Microstructural pathological alterations in DTI metrics in white matter in acromegaly. **(A)** Tract-based spatial statistics (TBSS) analysis of the DTI showed FA significantly decrease in widespread fiber bundles in acromegaly compared with controls (Blue, pTFCE < 0.05). **(B–D)** MD, RD, and AD significantly increase in widespread fiber bundles in acromegaly compared with controls (Red-yellow, pTFCE < 0.05).

DTI metrics, including FA, AD, MD, and RD, were also assessed in white matter using TBSS. As shown in **Figure 4A**, compared with the controls, acromegaly showed bilateral decreases in FA in many clusters across the mean FA skeleton. Inconsistent with reductions of FA, as shown in **Figures 4B–D**, AD, MD, and RD all showed widespread increases in many clusters across the tracts in the acromegaly compared with the controls.

### ROI Analysis

In line with the voxel-wise findings in GBSS, NDI in the cortex was significantly reduced in some ROIs in the acromegaly compared to the controls, including the caudate, cerebellum, insula, and putamen (**Table 2**). Consistent with the results of TBSS (**Table 3**), the ROI analysis showed significantly decreased NDI in the acromegaly relative to controls in 15 of 27 total tracts, including the left acoustic radiation, bilateral anterior thalamic radiation, bilateral cingulate gyrus part of cingulum, right corticospinal tract, forceps minor, bilateral inferior fronto-occipital fasciculus, middle cerebellar peduncle, bilateral superior longitudinal fasciculus, right superior thalamic radiation, and bilateral uncinate fasciculus. Besides, in some tracts, Viso was significantly increased in the acromegaly compared with controls. Interestingly, the ROI analysis found that ODI in the cortex, including in the insula and temporal lobes (**Table 2**), and ODI in the white matter in 11 of 27 total tracts was significantly decreased in the acromegalic patients compared with the controls (**Table 3**). Similarly, regarding the DTI metrics, compared with controls, the acromegaly showed significantly decreased FA in the bilateral anterior thalamic radiation, right cingulate gyrus part of cingulum, and forceps minor and significantly increased AD, RD, and MD in most tracts (**Supplementary Table 1**).

**Table 2:** NODDI metrics including NDI and ODI in cortex for ROI Analysis between acromegaly and controls.

Regions	NDI, Mean ± SD			ODI, Mean ± SD		
	Acromegaly	Controls	P	Acromegaly	Controls	P
Caudate	0.43 ± 0.02	0.45 ± 0.02	**.00**	0.44 ± 0.02	0.44 ± 0.01	.73
Cerebellum	0.58 ± 0.02	0.59 ± 0.02	**.00**	0.44 ± 0.01	0.44 ± 0.01	.72
Frontal Lobe	0.43 ± 0.02	0.44 ± 0.01	.11	0.48 ± 0.01	0.48 ± 0.01	.08
Insula	0.4 ± 0.02	0.42 ± 0.02	**.01**	0.44 ± 0.01	0.45 ± 0.01	**.00**
Occipital Lobe	0.44 ± 0.01	0.45 ± 0.01	.57	0.50 ± 0.01	0.50 ± 0.01	.13
Parietal Lobe	0.41 ± 0.01	0.41 ± 0.01	.76	0.49 ± 0.01	0.50 ± 0.01	.48
Putamen	0.52 ± 0.03	0.55 ± 0.03	**.00**	0.49 ± 0.02	0.49 ± 0.02	.92
Temporal Lobe	0.43 ± 0.02	0.44 ± 0.01	.08	0.47 ± 0.01	0.48 ± 0.01	**.00**
Thalamus	0.53 ± 0.04	0.53 ± 0.02	.55	0.37 ± 0.01	0.37 ± 0.01	.55

Group-differences are significant at a threshold of p < .05 using two-sample t-tests. Bold values mean there is a significant statistical difference.

**Table 3:** NODDI metrics including NDI, ODI, and Viso in white matter for ROI analysis between acromegaly and controls.

Tracts	NDI, Mean ± SD			ODI, Mean ± SD			Viso, Mean ± SD		
	Acromegaly	Controls	P	Acromegaly	Controls	P	Acromegaly	Controls	P
ar_l	0.57 ± 0.03	0.59 ± 0.02	**.01**	0.24 ± 0.01	0.24 ± 0.01	.35	0.09 ± 0.02	0.09 ± 0.02	.80
ar_r	0.57 ± 0.03	0.59 ± 0.03	.06	0.24 ± 0.01	0.24 ± 0.01	.15	0.09 ± 0.02	0.09 ± 0.02	.72
atr_l	0.60 ± 0.05	0.63 ± 0.02	**.00**	0.22 ± 0.01	0.22 ± 0.01	.45	0.09 ± 0.01	0.09 ± 0.01	.43
atr_r	0.59 ± 0.04	0.62 ± 0.03	**.00**	0.22 ± 0.01	0.22 ± 0.01	.89	0.08 ± 0.01	0.08 ± 0.01	.32
cgc_l	0.62 ± 0.04	0.64 ± 0.03	**.03**	0.14 ± 0.02	0.14 ± 0.02	.93	0.08 ± 0.01	0.08 ± 0.01	.96
cgc_r	0.59 ± 0.04	0.62 ± 0.03	**.01**	0.16 ± 0.02	0.16 ± 0.02	.55	0.08 ± 0.02	0.07 ± 0.01	**.01**
cgh_l	0.52 ± 0.05	0.53 ± 0.04	.48	0.26 ± 0.02	0.27 ± 0.02	.23	0.12 ± 0.04	0.13 ± 0.03	.55
cgh_r	0.51 ± 0.05	0.53 ± 0.03	.16	0.26 ± 0.03	0.26 ± 0.02	.77	0.12 ± 0.04	0.13 ± 0.04	.75
cst_l	0.68 ± 0.03	0.69 ± 0.02	.09	0.16 ± 0.01	0.17 ± 0.01	.35	0.11 ± 0.01	0.11 ± 0.01	.11
cst_r	0.68 ± 0.03	0.69 ± 0.02	**.02**	0.16 ± 0.01	0.16 ± 0.01	.08	0.11 ± 0.01	0.11 ± 0.01	.79
fma	0.60 ± 0.03	0.62 ± 0.02	.09	0.12 ± 0.01	0.13 ± 0.01	**.02**	0.13 ± 0.02	0.12 ± 0.02	.14
fmi	0.58 ± 0.04	0.63 ± 0.03	**.00**	0.16 ± 0.01	0.17 ± 0.01	**.00**	0.10 ± 0.01	0.11 ± 0.02	.11
ifo_l	0.55 ± 0.04	0.57 ± 0.02	**.01**	0.16 ± 0.01	0.17 ± 0.01	**.01**	0.09 ± 0.01	0.09 ± 0.01	.13
ifo_r	0.55 ± 0.04	0.58 ± 0.03	**.00**	0.16 ± 0.01	0.17 ± 0.01	**.02**	0.09 ± 0.01	0.09 ± 0.01	.88
ilf_l	0.52 ± 0.04	0.54 ± 0.03	.07	0.16 ± 0.01	0.17 ± 0.01	**.00**	0.08 ± 0.01	0.07 ± 0.01	**.04**
ilf_r	0.52 ± 0.04	0.54 ± 0.03	.05	0.15 ± 0.01	0.16 ± 0.01	**.00**	0.08 ± 0.01	0.08 ± 0.01	.48
mcp	0.78 ± 0.04	0.80 ± 0.03	**.04**	0.20 ± 0.03	0.20 ± 0.02	.72	0.21 ± 0.04	0.19 ± 0.04	**.03**
ml_l	0.66 ± 0.04	0.67 ± 0.03	.60	0.20 ± 0.01	0.20 ± 0.01	.12	0.15 ± 0.02	0.17 ± 0.02	.06
ml_r	0.66 ± 0.05	0.67 ± 0.03	.56	0.20 ± 0.02	0.20 ± 0.01	.13	0.16 ± 0.02	0.17 ± 0.02	.20
ptr_l	0.53 ± 0.04	0.54 ± 0.02	.31	0.18 ± 0.01	0.19 ± 0.01	**.00**	0.08 ± 0.02	0.08 ± 0.01	.08
ptr_r	0.54 ± 0.04	0.55 ± 0.03	.21	0.18 ± 0.01	0.18 ± 0.01	**.02**	0.09 ± 0.01	0.09 ± 0.01	.28
slf_l	0.64 ± 0.04	0.66 ± 0.03	**.03**	0.17 ± 0.01	0.17 ± 0.01	**.04**	0.09 ± 0.01	0.08 ± 0.01	**.02**
slf_r	0.62 ± 0.04	0.65 ± 0.03	**.00**	0.18 ± 0.02	0.18 ± 0.02	.83	0.08 ± 0.01	0.08 ± 0.01	.64
str_l	0.65 ± 0.04	0.66 ± 0.03	.21	0.20 ± 0.01	0.21 ± 0.01	.16	0.09 ± 0.01	0.08 ± 0.01	.06
str_r	0.64 ± 0.03	0.66 ± 0.02	**.03**	0.20 ± 0.01	0.21 ± 0.01	**.04**	0.09 ± 0.01	0.08 ± 0.01	**.03**
unc_l	0.51 ± 0.04	0.54 ± 0.03	**.00**	0.20 ± 0.02	0.21 ± 0.02	**.01**	0.07 ± 0.02	0.07 ± 0.01	.64
unc_r	0.50 ± 0.04	0.53 ± 0.03	**.00**	0.20 ± 0.01	0.20 ± 0.01	.27	0.06 ± 0.01	0.06 ± 0.01	.63

l, left; r, right; ar, acoustic radiation; atr, anterior thalamic radiation; cgc, cingulate gyrus part of cingulum; cgh, parahippocampal part of cingulum; cst, corticospinal tract; fma, forceps major; fmi, forceps minor; ifo, inferior fronto-occipital fasciculus; ilf, inferior longitudinal fasciculus; mcp, middle cerebellar peduncle; ml, medial lemniscus; ptr, posterior thalamic radiation; slf, superior longitudinal fasciculus; str, superior thalamic radiation; unc, uncinate fasciculus. Group-differences are significant at a threshold of p < .05 using two-sample t-tests. Bold values mean there is a significant statistical difference.

### Correlations Between Imaging Parameters and Clinical Features

**Figure 5:**
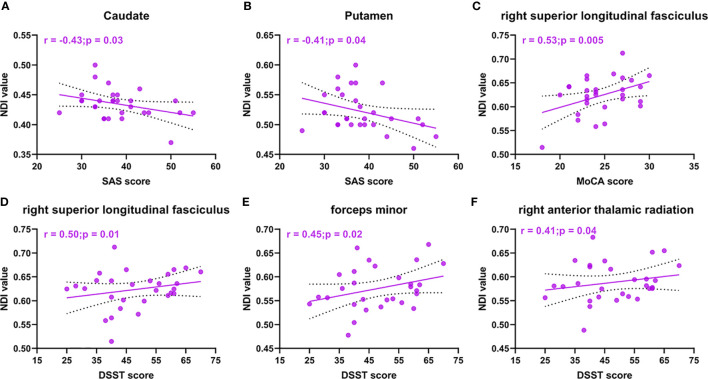
NODDI metrics correlations with neuropsychological disorders in acromegaly. Cortical NDI in caudate **(A)** and putamen **(B)** are negative correlation with SAS score. NDI in right superior longitudinal fasciculus are positive correlation with MoCA score **(C)** and DSST score **(D)**. NDI in forceps minor **(E)** and right anterior thalamic radiation **(F)** were positively correlated with DSST score.

In our study, we found that the patients with acromegaly had more neuropsychological disorders than the controls. Thus, we further analyzed whether there were potential relationships between neuropsychological disorders and microstructural pathological changes in the acromegalic patients based on the ROI analysis results with NODDI and DTI metrics, while correcting for covariance (age and education). In the cortex for NODDI metrics, as shown in **Figures 5A, B**, we found that the NDI in both the caudate (r = -0.43, p = 0.03) and putamen (r = -0.41, p = 0.04) were negatively correlated with SAS score. In white matter for NODDI metrics, as shown in **Figures 5C–F**, both MoCA (r = 0.53, p = 0.005) and DSST (r = 0.50, p = 0.01) were positively correlated with NDI in the right superior longitudinal fasciculus and NDI in the forceps minor (r = 0.45, p = 0.02) and right anterior thalamic radiation (r = 0.41, p = 0.04) were positively correlated with DSST. In addition, in white matter for DTI metric, the results also showed both MoCA and DSST score were negatively correlated with MD and RD in the right superior longitudinal fasciculus (**Supplementary Figure 1**). FA in the forceps minor and right superior thalamic radiation, MD in the right anterior thalamic radiation and right superior thalamic radiation, and RD in the forceps minor and right anterior thalamic radiation were correlated with DSST score (**Supplementary Figure 1**).

In addition, we also investigated the potential relationships between serum excess GH/IGF-1 levels and microstructural pathological changes in acromegaly. However, myelin imaging, NODDI and DTI metrics did not show any significant associations with serum GH/IGF-1 levels (TFCE corrected, p < 0.05).

## Discussion

GH and IGF-1 play multiple important roles in the CNS. To date, however, the impact of long-term and persistent excess serum GH/IGF-1 levels on cortical and white matter microstructures, such as myelin and neural fibers, has largely remained unknown in acromegaly. To the best of our knowledge, this is the first study to provide an *in vivo* characterization of cortical and white matter microstructural pathology in acromegaly by SBM, T1/T2-weight ratio, NODDI and DTI analyses.

In our study, we found widespread increases in cortical thickness in the patients with acromegaly compared to the controls with SBM analysis. This result is consistent with our previous voxel-based morphometry (VBM) analysis, which showed a diffuse increase in GM volume (17). The diffuse increase in cortical thickness may be related to the role of GH/IGF-1 in contributing to the proliferation of cells in the CNS (10, 30). Previous studies have shown that GH and IGF-1 play important roles in the formation of the myelin sheath (10, 30). In this study, T1/T2-weighted myelin-sensitive MRI was used to detect myelin microstructure alterations in acromegaly with long-term persistent serum excess GH/IGF-1 levels. Both *ex vivo* and *in vivo* studies indicated that lower T1/T2-weighted ratio reflects demyelination in cortex and white matter (31, 32). In the cortex, we found that T1/T2-weighted ratio was significantly decreased in the bilateral occipital cortex and pre/postcentral gyri but were significantly increased T1/T2-weight ratio in the bilateral fusiform, bilateral insular, and bilateral superior temporal gyri in acromegaly compared with the controls. In the white matter, our results showed that T1/T2-weighted ratio was widespread decreases, with only a few areas showing increased T1/T2-weighted ratio in acromegaly compared with controls. These results were consistent with the observed demyelination in the PNS in patients with acromegaly in previous studies (33, 34). The increase in T1/T2-weighted ratio in some GM and WM regions may be related to the formation of the myelin sheath in these regions present under persistent excess serum GH/IGF-1 levels because the distribution of GH/IGF-1 receptors is dissimilar across brain regions (35, 36). However, correlation analysis showed that no significant negative correlation was observed between the higher GH/IGF-1 levels and T1/T2-weighted ratio. Taken together, our results suggested that long-term and persistent excess serum GH/IGF-1 levels can lead to extensive demyelination and remyelination in the cortex and white matter in acromegaly. This polarization result may be related to the obvious differences about the distribution of GH/IGF-1 receptors in brain regions, but this hypothesis needs future animal experiments or anatomy research to confirm.

To date, NODDI has been applied to several neurologic and psychiatric conditions, including multiple sclerosis, Alzheimer’s disease, amyotrophic lateral sclerosis, Parkinson’s disease, and Huntington’s disease, as it provides specific microstructural insights in both the cortex and white matter (31, 37). Both ex vivo and *in vivo* studies have suggested that decreases in NDI reflect the loss of neurite integrity and that increases in ODI reflect the loss of fibre coherence in the cortex and white matter (38, 39). In the current study, we also used NODDI to detect microstructural pathological changes in both the cortex and white matter in acromegaly. Our data indicated that NDI showed widespread decreases in the cortex in the patients with acromegaly. Similarly, in white matter, we also observed significant reductions in NDI in almost all fiber bundles in the patients with acromegaly compared to the controls. However, there were no significant differences in ODI between the two groups. In addition, we also found that Viso was significantly increased in the WM fiber bundles in acromegaly relative to controls, which indicated that increased extracellular fluid in the white matter was caused by long-term excessive hormone levels. In our previous study, we found diffuse increases in WM volume in patients with acromegaly (17). This may be due to the increase in extracellular fluid in white matter. Further animal experiments or autopsy are needed to find out why acromegalic patients have diffuse increases in WM volume while most of the fiber tracts showed demyelinating changes. These NODDI findings were further supported by our independent DTI analysis, where FA, AD, RD and MD were calculated through TBSS. Together with the results of NODDI, we found that decreased FA and increased MD, RD and AD were displayed in extensive white matter fiber bundles. Lower FA and higher MD, RD and AD have been used as surrogate measures of microstructural changes of tissue, such as demyelination, axonal degeneration, axonal damage, and axonal loss. Furthermore, entire functional cortical regions and fiber bundle microstructural alterations were investigated using ROI analyses in NODDI and DTI metrics to cross-examine microstructural pathological alterations in specific brain regions and fiber bundles caused by long-term and persistent excess serum GH/IGF-1 levels in acromegaly. Our results showed that most functional cortical regions and fibre bundles had microstructural pathological alterations in the acromegalic patients compared with that controls, which indicated that long-term and persistent excess serum GH/IGF-1 levels play a vital role in adult brain. Interestingly, we also found decreases in cortical ODI, including in the insula and temporal lobe, and decreases in ODI in the white matter in 11 of 27 total tracts in the acromegalic patients compared with the controls. However, no relationships between serum GH/IGF-1 levels and parameters of NODDI and DTI were found. This may have been due to fluctuations in GH/IGF-1 levels during disease development, as well as the length of time that GH/IGF-1 acts on the brain. In summary, our results suggested that there was widespread microstructural pathology in both the cortex and white matter in acromegaly under long-term and persistent excess serum GH/IGF-1 levels. In animal with transgenic IGF-I overexpression, researchers found that IGF-1 and GH can promote neurogenesis and increase myelin content (40). But in this study, we found extensive destruction of nerve fiber and myelin integrity. This may suggest that high GH/IGF-1 level may exert unique effects during different stages of the lifespan.

Neuropsychological dysfunction often occurs in patients with acromegaly (3). However, little is known about its mechanism. In the current study, we also found that the acromegalic patients performed worse than the controls in MoCA, DSST, and SAS tests. In the significantly altered ROIs based on the NODDI and DTI metrics, we found decreases in NDI in the caudate and putamen were associated with SAS score. The caudate and putamen are important components of striatal brain regions, which are included in the anxiety network (41). Our findings suggested that microstructural pathology in the caudate and putamen may be the mechanism of anxiety in acromegaly. The MoCA test provides information about disturbances in general cognitive functioning. In addition, The DSST is sensitive to various cognitive processes including processing speed, sustained attention, visual spatial skills, set shifting, and working memory (42). The MoCA supplemented by the DSST has been suggested to be more effective in screening for cognitive impairment in type 2 diabetes mellitus (43). In significantly altered ROIs based on NODDI and DTI metrics, we found microstructural pathological alterations including NDI, MD and RD in the right superior longitudinal fasciculus (SLF) were associated with MoCA and DSST score. The SLF, encompassing multiple cognitive functions such as attention, language, visual-spatial skills, memory, and executive function, is a massive bundle and a bidirectional pathway that mainly connects the frontal lobe cortex to the parietal, temporal, and occipital lobe cortices (44, 45). Some studies had previously reported that, in humans, microstructural pathology in the right SLF was associated with cognitive impairment in neurological diseases (46, 47). In addition, we found microstructural pathological alterations, including NDI, FA, and RD, in the forceps minor were associated with DSST score. The forceps minor is part of the fiber radiation of the corpus callosum that bends forward towards the frontal pole of the cerebrum and is considered a substantial circuit connecting the bilateral regions of the anterior default mode network (48). Previous studies have already reported that microstructural pathology in the forceps minor was responsible for cognitive impairments (49, 50). Our findings suggest that microstructural pathology in cortex and white matter may be responsible for the neuropsychological dysfunction in patients with acromegaly.

There are some limitations in the current study. The MoCA and DSST implemented in the present study are well-established to measure global cognition, attention, and memory, but more specific and targeted neuropsychological test batteries, such as spatial navigation/virtual environment tasks, and the Wechsler Memory Scale, would help to shed more light on the detailed cognitive impairment patterns in those with acromegaly and investigate their potential relationships with microstructural pathological alterations. In addition, longitudinal imaging data from the patients with acromegaly after surgery are lacking to further investigate whether there are dynamic microstructural alteration in the cortex and white matter in acromegalic patients with decreases serum GH/IGF-1 levels. Finally, this study enrolled a small number of acromegalic patients, and a larger number of acromegalic patients enrolled may be acquire the model of different high serum GH/IGF-1 levels on the grey matter and white matter microstructural pathologies.

In conclusion, our findings demonstrated that long-term and persistent excess serum GH/IGF-1 levels alter microstructure in cortex and white matter in acromegaly. These microstructural pathologies may be responsible for the neuropsychological dysfunction in acromegaly.

## Data Availability Statement

The datasets generated for this study are available on request to the corresponding authors.

## Ethics Statement

The studies involving human participants were reviewed and approved by Beijing Tiantan Hospital Ethics Committee. The patients/participants provided their written informed consent to participate in this study.

## Author Contributions

Designed and conceptualized study (TY, YZ). Major role in the acquisition of data (JY, CZL, LJ, JK, YS, ZZ, SG, RW, CHL). Analyzed the data (ZZ, TY, RW). Drafted the manuscript for intellectual content (TY, ZZ, YZ). All authors contributed to the article and approved the submitted version.

## Funding

This study was supported by the Ministry of Science and Technology of China Grant (2019FYA0707103 and 2015AA020504), the National Nature Science Foundation of China Grant (81771489, 31730039, and 81871350), the Beijing Municipal Science & Technology Commission Grant (Z171100000117002), and the Strategic Priority Research Program of Chinese Academy of Sciences (XDB32010300).

## Conflict of Interest

The authors declare that the research was conducted in the absence of any commercial or financial relationships that could be construed as a potential conflict of interest.
