# Supplementary material for: In Vivo Characterization of Cortical and White Matter Microstructural Pathology in Growth Hormone-Secreting Pituitary Adenoma
Source: Front Oncol. 2021 Apr 12;11:641359. doi: 10.3389/fonc.2021.641359 (PMC8072046; doi:10.3389/fonc.2021.641359)
Supplement: Supplementary file 1 [file DataSheet_1.pdf]

Supplementary Table1. DTI metrics including FA, MD, AD, and RD for ROI analysis between acromegaly and controls.

	FA, Mean ± SD			MD, Mean ± SD			AD, Mean ± SD			RD, Mean ± SD		
	Acrome	Controls	P	Acrome	Controls	P	Acrome	Controls	P	Acrome	Controls	P
ar_l	0.46±0.02	0.47±0.02	.05	0.61±0.02	0.6±0.02	**.01**	0.94±0.02	0.92±0.02	**.03**	0.45±0.02	0.43±0.02	**.01**
ar_r	0.47±0.02	0.47±0.02	.21	0.6±0.02	0.59±0.02	.13	0.92±0.02	0.92±0.02	.26	0.44±0.02	0.43±0.02	.15
atr_l	0.46±0.03	0.48±0.02	**.01**	0.58±0.03	0.56±0.01	**.00**	0.9±0.03	0.88±0.02	**.02**	0.42±0.03	0.4±0.02	**.00**
atr_r	0.46±0.03	0.48±0.02	**.01**	0.59±0.02	0.57±0.01	**.00**	0.9±0.03	0.89±0.02	**.01**	0.43±0.03	0.4±0.02	**.00**
cgc_l	0.58±0.03	0.59±0.03	.23	0.58±0.02	0.57±0.02	**.02**	1.04±0.03	1.02±0.03	.10	0.36±0.03	0.35±0.03	.07
cgc_r	0.54±0.03	0.55±0.03	**.04**	0.59±0.02	0.57±0.02	**.00**	1.00±0.03	0.98±0.03	**.03**	0.39±0.03	0.37±0.03	**.01**
cgh_l	0.38±0.03	0.38±0.02	.79	0.67±0.03	0.66±0.03	.37	0.95±0.04	0.94±0.03	.16	0.53±0.03	0.53±0.03	.62
cgh_r	0.39±0.03	0.39±0.03	.47	0.67±0.03	0.66±0.03	.13	0.97±0.04	0.95±0.03	.14	0.53±0.03	0.52±0.03	.21
cst_l	0.59±0.02	0.59±0.02	.76	0.54±0.02	0.53±0.01	.05	0.95±0.03	0.94±0.03	.13	0.33±0.02	0.33±0.01	.16
cst_r	0.59±0.02	0.59±0.02	.64	0.54±0.01	0.54±0.01	.07	0.96±0.03	0.95±0.02	.21	0.34±0.02	0.33±0.01	.15
fma	0.63±0.02	0.63±0.02	.48	0.62±0.02	0.61±0.02	**.03**	1.15±0.03	1.13±0.03	.08	0.35±0.02	0.34±0.02	.10
fmi	0.55±0.03	0.57±0.02	**.02**	0.6±0.03	0.57±0.02	**.00**	1.03±0.03	0.99±0.02	**.00**	0.39±0.03	0.37±0.02	**.00**
ifo_l	0.52±0.02	0.52±0.02	.65	0.62±0.03	0.6±0.01	**.00**	1.02±0.04	0.99±0.02	**.00**	0.42±0.03	0.4±0.02	**.04**
ifo_r	0.52±0.03	0.53±0.02	.13	0.62±0.03	0.6±0.02	**.00**	1.02±0.03	1.00±0.02	**.00**	0.41±0.03	0.4±0.02	**.02**
ilf_l	0.52±0.02	0.52±0.02	.33	0.63±0.02	0.62±0.02	**.00**	1.04±0.04	1.01±0.02	**.00**	0.43±0.02	0.42±0.02	.18
ilf_r	0.53±0.02	0.53±0.02	.93	0.63±0.03	0.62±0.02	**.03**	1.05±0.03	1.03±0.02	**.00**	0.43±0.03	0.42±0.02	.22
mcp	0.56±0.03	0.57±0.02	.24	0.53±0.02	0.51±0.02	**.00**	0.91±0.04	0.89±0.04	**.04**	0.34±0.02	0.33±0.02	**.01**
ml_l	0.51±0.02	0.51±0.01	.37	0.57±0.02	0.58±0.02	.17	0.93±0.04	0.95±0.03	.14	0.39±0.02	0.4±0.01	.32
ml_r	0.51±0.02	0.51±0.02	.78	0.57±0.03	0.58±0.01	.40	0.94±0.04	0.94±0.02	.35	0.39±0.02	0.4±0.01	.58
ptr_l	0.51±0.03	0.5±0.02	.20	0.63±0.02	0.62±0.02	.06	1.03±0.03	1.00±0.02	**.00**	0.43±0.03	0.43±0.02	.78
ptr_r	0.51±0.03	0.51±0.02	.76	0.63±0.02	0.62±0.02	.13	1.03±0.03	1.01±0.02	**.00**	0.43±0.03	0.42±0.02	.64
slf_l	0.55±0.02	0.56±0.02	.17	0.54±0.02	0.53±0.02	**.01**	0.91±0.02	0.9±0.02	**.01**	0.36±0.02	0.35±0.02	**.02**
slf_r	0.53±0.03	0.54±0.03	.05	0.55±0.02	0.53±0.02	**.00**	0.91±0.03	0.89±0.03	**.02**	0.37±0.03	0.36±0.02	**.01**
str_l	0.53±0.02	0.53±0.02	.89	0.54±0.02	0.54±0.01	.08	0.9±0.03	0.88±0.02	.05	0.37±0.02	0.36±0.02	.30
str_r	0.52±0.02	0.52±0.02	.56	0.55±0.02	0.54±0.01	**.02**	0.89±0.03	0.88±0.02	.05	0.37±0.02	0.36±0.01	.06
unc_l	0.45±0.03	0.46±0.02	.32	0.64±0.03	0.62±0.02	**.00**	0.99±0.03	0.95±0.02	**.00**	0.47±0.03	0.45±0.02	**.00**
unc_r	0.46±0.02	0.47±0.02	.08	0.64±0.02	0.62±0.02	**.00**	0.99±0.03	0.97±0.02	**.00**	0.47±0.03	0.45±0.02	**.01**

Legend: Acrome = acromegaly, l = left, r = right, ar = acoustic radiation, atr = anterior thalamic radiation, cgc = cingulate gyrus part of cingulum, cgh = parahippocampal part of cingulum, cst = corticospinal tract, fma = forceps major, fmi = forceps minor, ifo = inferior fronto-occipital fasciculus, ilf = inferior longitudinal fasciculus, mcp = middle cerebellar peduncle, ml = medial lemniscus, ptr = posterior thalamic radiation, slf = superior longitudinal fasciculus, str = superior thalamic radiation, unc = uncinate fasciculus. Group-differences are significant at a threshold of $p < .05$ using two-sample t-tests.

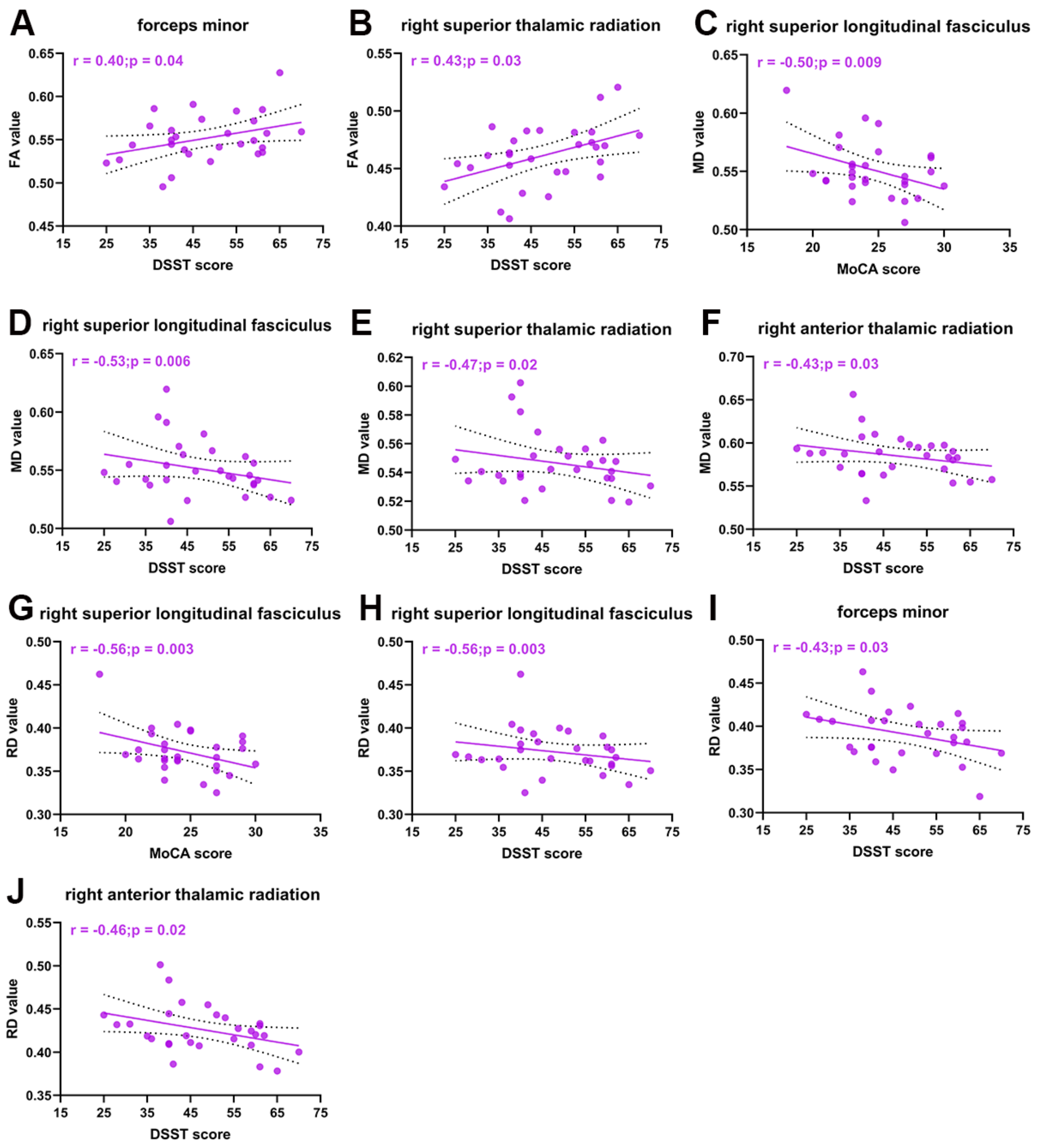

Supplementary Figure 1. DTI metrics correlations with neuropsychological disorders in acromegaly. FA in forceps minor (A) and right superior thalamic radiation (B) were positively correlated with DSST score. MD in right superior longitudinal fasciculus are negative correlation with MoCA score (C) and DSST score (D). MD in right superior thalamic radiation (E) and right anterior thalamic radiation (F) are negative correlation with DSST score. RD in right superior longitudinal fasciculus are negative correlation with MoCA score (G) and DSST score (H). RD in forceps minor (I) and right anterior thalamic radiation (J) were negatively correlated with DSST score.
